# 

**Published:** 1884-01

**Authors:** 


ORIGINAL COMMUNICATIONS:
A Day's Practice. N. S. Jenkins.... 1
A Reply to some Views on the Putrefactive Theory of Decay. W. D. Miller.... 15
Relation of Food to the Teeth. E. C. Kirk.... 21
A New Method of Filling Teeth. C. A. Timme.... 27
The Use and Abuse of Gold as a Filling Material. A. N. Roussel.... 29

REPORTS OF SOCIETY MEETINGS:
Connecticut Valley Dental Society.... 32

CORRESPONDENCE:
A Warning to the Profession.... 38
Pre-Historic Teeth.... 40
Gum Guillotine Forceps.... 43
Pre-Historic Dental Diseases.... 44

EDITORIAL:
The Missouri Dental Journal.... 45
Dental Societies.... 47
Ancient Peruvians.... 50
"Index Medicus".... 50
"Abbott's Scissors".... 51
August Number Wanted.... 51
Deferred.... 51

CURRENT NEWS AND OPINION:
Personal.... 20
Bogus Diplomas.... 28
German Graduates.... 51
University of California.... 52
Dental Legislation.... 52
New York Odontological Society.... 53
Plaster Models.... 53
Popular Science News.... 54
Good Teeth and Good Intellect.... 54
Declined with Thanks.... 54
Resigned.... 54
Trachetomy for the Extraction of a Tooth from the Left Bronchus.... 55
Hay Fever.... 55
Carbolic Acid.... 55
In India.... 55

